# The relationship between learning organization system on innovation performance: testing moderated mediation role of organizational cultural identification

**DOI:** 10.3389/fpsyg.2026.1828291

**Published:** 2026-04-17

**Authors:** Han Cai, Jiaxi Wang, Xiu Jin

**Affiliations:** 1 Department of Cross-border E-commerce, School of Management, Liaodong University, Dandong, China; 2 Department of Culture and Art Management, Honam University, Gwangju, Republic of Korea; 3 Department of Business Administration, Gachon University, Seongnam, Republic of Korea

**Keywords:** innovation performance, knowledge sharing, learning organization system, organization system, organizational cultural identification

## Abstract

The organizational growth and sustainability are closely related to how well the organizational system is functioning. In particular, the organizational innovation and competitiveness are related to the creativity and innovation through knowledge sharing among organizational members in a competitive situation. In relation to such background, this study focused on the learning organization system as a key variable that drives to knowledge sharing among organizational members. As a product of the knowledge economy era, the learning organization system plays a crucial role in an organization’s development process. The establishment of a learning organization system helps create a shared learning platform, which reduces the cost of acquiring knowledge for employees, promotes joint learning among employees, and effectively facilitates the rapid dissemination of knowledge within the organization. This practice effectively stimulates employees’ innovation ability and enhances their innovation performance, which, in turn, provides important support for the sustainable development of organizations. Therefore, this study argues that organizational learning systems are closely related to employees’ innovation performance. In this context, this study investigates whether learning organizational systems can improve employees’ innovation performance and the mediating role of employees’ knowledge sharing behaviors in promoting innovation performance. Most previous studies focused only on the mediating or moderating roles of the model. This study expands the research field by examining the moderating role of organizational cultural identification. We also verified its moderating role. Overall, this study aims to determine improvements in the innovation performance of employees under a learning organization system. Based on this, suggestions are proposed to strengthen and optimize the learning organization system and provide a theoretical basis for related research. This study collected survey data from 300 Chinese SME employees for empirical analysis. The results show that the learning organization system has a positive effect on employee innovation performance. Knowledge sharing can also positively influence innovation performance. In addition, the interaction between organizational cultural identification and the learning organizational system improves employees’ knowledge sharing. Overall, we contributed to the field of learning organization system research and fill the theoretical and practical gaps in existing related research.

## 1 Introduction

In the current era of rapid information ionization, enterprises face unprecedented data growth and knowledge challenges (Zhou et al., 2024). In this competitive environment, whether firms can realize sustainable growth depends on their innovation capabilities (Xiong and Tao, 2024). The knowledge factor, as an important new type of factor, has replaced the traditional innovation factor and is crucial for the innovation and performance improvement of enterprises. Learning organizations are prerequisites for organizational transformation and performance improvement, and the establishment of systematization further supports this transformation process. Because a learning organization system is an environment that facilitates the development of individual and organizational learning (Elkjaer and Wahlgren, 2005), it aims to provide a common learning platform and knowledge sharing mechanism for organizational members, who play a key role in the organization. Under the learning organization system, employees share common interests and goals and are more willing to improve their performance in the organization through the transfer of information and make efforts toward the sustainable development of the organization (Wiyana and Sriathi, 2021). Moreover, the creation and formation of a learning organizational system can encourage employees to improve their performance and help in the development of the organization (Joo, 2012). In addition, a learning organization system can develop employees with high learning abilities (Purwanti and Rizky, 2012), transfer the correct core values and beliefs of the organization to employees, and improve their continuous innovation to contribute to the sustainable development of the organization (Yu, 2007). Therefore, these highlights emphasize the key role of the learning organization system in organizational management. We should commit to building and continuously improving such a system to support employee learning and lay a solid foundation for future competitive advantage.

The emergence of the learning organization system as an organizational management system is aimed at enhancing the management quality of the enterprise, strengthening the continuous learning of employees within the organization, and enhancing the cohesion of the organization (Yu, 2007), helping employees to communicate effectively with each other and share their experiences within the organization, thus enhancing the learning ability of employees and stimulating innovation (Chen, 2008). The establishment of a learning organization enhances learning ability within the organization, which has a positive impact on innovation performance (Farooq, 2012). Moreover, innovation performance is an expression of the efficiency and effectiveness of an organization in carrying out innovative activities, and the improvement of innovation performance is essential to increase the value of the organization and enhance its competitive advantage (Wang, 2020). Specifically, employees’ success relies on a learning organization system that provides access to opportunities to learn and practice new things and skills, and employees further increase their self-knowledge and experience through continuous learning and change, innovation, and raising the level of their performance (Ambarwati et al., 2020). Therefore, this study predicts that a learning organization system will strengthen the innovation performance of employees, thus contributing to the future growth of the organization.

This study aims to determine whether a learning organization system will help enhance employees’ innovation performance and thus increase the level of innovation performance. It also suggests that knowledge sharing has a mediating effect on the relationship between the learning organization system and innovation performance. From the perspective of social exchange theory, employees’ knowledge sharing behaviors are premised on their access to relevant resources and needs in the organization (Li and Qi, 2023). In other words, a learning organization system not only emphasizes individual learning but also fosters mutual learning among employees, helping them recognize shared interests and become more willing to share knowledge, experiences, and lessons to achieve a common vision (Chen, 2008). As employees disseminate and apply knowledge within an organization, it can foster innovation, improve decision-making, and promote collective intelligence for sustainable organizational development (Zamiri and Esmaeili, 2024). Specifically, the practice of learning organizational systems will not only increase the autonomy of employees and help them learn new skills but also promote the sharing of knowledge among employees in the organization, which will motivate them to improve their innovation performance by continuously innovating to improve their processes (Laeeque et al., 2017). Therefore, this study concludes that a learning organization system will impact innovation performance through knowledge sharing among employees.

In addition, we need to examine a moderating variable to validate its effect on the learning organization system and test its interaction with the learning organization system to further understand its effect on knowledge sharing. In this process, we focus on organizational cultural identification and argue that the level of knowledge sharing among employees’ changes with the moderation of organizational cultural identification. Organizational cultural identification is an employee’s attitude toward organizational culture, which is formed through comparison and screening in the process of interacting with the organization. It is a process of understanding organizational culture from shallow to deep (Han and Wang, 2015). Employees’ organizational cultural identification can promote a stronger conception of the direction of organizational development and inspire them to support the realization of organizational culture through practical actions (Ding and Luo, 2012). Social exchange theory posits that there is an interactive and mutually influential relationship between individual behavior and the social environment (Mustika et al., 2020). Employees who identify with organizational values view its efforts as meaningful, making them more likely to align their behaviors with group direction and in return, organizational recognition and motivation encourage them to fulfill their potential and contribute more (Han and Wang, 2015). In addition, the organization will form a learning organizational culture internally through the learning system, promote employees’ learning and knowledge accumulation, motivate employees, and encourage innovation. In such an environment, employees feel that their value has been enhanced, thus increasing their organizational cultural identification (Lingtao, 2023). Moreover, they are more likely to actively participate in learning and knowledge sharing activities in this situation, which enhances their motivation and sense of belonging and prompts them to invest in the learning and development of the organization. This study concludes that the learning organization system and organizational cultural identification are mutually reinforcing and together constitute a virtuous circle within the organization, laying the foundation for sustainable organizational development. Therefore, it is necessary to explore the moderating effect of organizational cultural identification and argue that the interaction between the learning organization system and organizational cultural identification increases knowledge sharing among employees.

Based on the above theories, the objectives of this study are summarized as follows: First, previous studies indicate that research on learning organization systems remains at an initial stage, with a notable scarcity of empirical research examining the relationship between learning organization systems and innovation performance. Although existing literature has acknowledged the conceptual relevance of learning organization systems to organizational outcomes, relatively few studies have empirically tested how such systems translate into employees’ individual innovation performance. To address this gap, this study not only clarifies the relationship between learning organization systems and innovation performance but also elucidates the underlying mechanisms through which learning organization systems drive innovation performance. Therefore, this study contributes to advancing the theoretical understanding of learning organization systems and provides empirical evidence that helps extend the current literature beyond conceptual discussions toward a more rigorous examination of its performance implications.

Second, although most recent studies have focused on learning organizations as an effective organizational form (Fan, 2003), they have largely overlooked the concept of learning organization systems. Unlike the static notion of a learning organization, a learning organization system reveals environmental relationships and system feedback—encompassing organizational structure, culture, and operation mode—thereby enabling environments to interact and adapt, keeping the organization in a dynamic process of continuous learning and change (Zhang and Cui, 2008). However, existing literature has treated these two concepts as interchangeable, neglecting the distinct mechanisms through which learning organization systems, as an integrated systemic framework, influence organizational and employee outcomes. Therefore, to address this issue, this study shifts the analytical focus from learning organizations as an organizational form to learning organization systems as a dynamic management mechanism. In doing so, it provides evidence on how the systemic features of learning organization systems—rather than merely the presence of a learning organization—drive employee innovation performance. Consequently, this study contributes to the literature by offering a more nuanced understanding of learning organization systems and establishing a foundation for future research to explore their multidimensional effects on organizational behavior.

Third, while most existing studies have treated organizational cultural identification solely as an independent or mediating variable, they have overlooked its potential as a boundary condition that may shape the effectiveness of organizational systems. This limited perspective fails to capture the nuanced role of organizational cultural identification in moderating the relationship between organizational mechanisms and employee behaviors. To address this issue, this study identifies and explores the moderating role of organizational cultural identification. Specifically, it examines how the interaction between learning organization systems and organizational cultural identification influences employees’ knowledge-sharing behaviors. This study extends the current understanding of organizational cultural identification beyond its direct effects, identifying it as a critical factor that amplifies the effectiveness of learning organization systems. This contribution not only enriches the theoretical framework of organizational cultural identification but also provides practical insights for organizations seeking to leverage cultural identification to enhance knowledge-sharing outcomes.

Finally, despite the growing recognition of learning organization systems in organizational research, existing studies have largely overlooked their application in the context of Chinese small and medium-sized enterprises, leaving a critical gap in understanding how such systems function within this unique institutional and cultural setting. Moreover, the role of organizational systems in shaping employee outcomes remains underexplored and undertheorized. To address these gaps, this study proposes a new research paradigm that empirically investigates how learning organization systems enhance employee innovation performance, thereby expanding the research field of learning organization systems and innovation performance. Specifically, this study reveals the importance of organizational systems in Chinese SMEs at the current stage and elucidates the role of organizational cultural identification in shaping knowledge-sharing behaviors through its interaction with learning organization systems. By doing so, this research not only extends the theoretical boundaries of learning organization systems but also provides contextualized insights that contribute to both academic understanding and practical implications for Chinese SMEs navigating organizational system implementation.

## 2 Theoretical background and hypotheses

### 2.1 Learning organization system and knowledge sharing

A learning organization system is a management system that consciously and systematically leverages knowledge resources to adapt to internal and external changes, thereby sustaining competitive advantage (Chen, 2002). It also refers to a management system that can create a creative atmosphere that leads to increased competence and provides the basis for the organization to achieve superior performance (Wiyana and Sriathi, 2021). The emergence of a learning organization system positively affects organizational effectiveness by helping to increase the satisfaction of the organization’s employees and contributing to the improvement of organizational performance (Chen, 2008).

According to prior research, learning organizational systems have a significant positive impact on a company’s business (Purwanti and Rizky, 2012), employee innovation ability (Fanbasten, 2014), and knowledge sharing (Laksono, 2023).

Knowledge sharing is the activity of acquiring and facilitating the transfer of knowledge to others (Eletter et al., 2022). Knowledge sharing allows employees to realize the potential value of knowledge, enabling organizations to identify valuable insights that enhance processes and drive organizational change (Zhu et al., 2023). Knowledge sharing is the process of exchanging ideas, insights, experiences, and specialized knowledge between individuals or groups (Zamiri and Esmaeili, 2024). Through knowledge sharing behaviors, employees have better access to the required knowledge, which enhances innovation performance for the future development of the organization (Wan and Chen, 2012). According to prior research, knowledge-sharing behavior has a positive impact on innovation performance (Zhu et al., 2023), innovative job performance (Singh et al., 2021), and workplace ostracism inhibits the occurrence of employee knowledge sharing behavior (Li and Qi, 2023).

Learning organizations advocate the exchange of information between all levels of the organization, and the establishment of a learning organization system helps develop organizational memory, thus facilitating the development of knowledge sharing (Pan et al., 2020). A learning organization system enables employees to recognize their interconnection and adopt a holistic organizational perspective, thereby promoting collaboration, knowledge dissemination, and knowledge-sharing behaviors through mutual learning (Fanbasten, 2014). A learning organization system raises employees’ awareness of organizational learning and knowledge sharing, facilitating knowledge flow between groups and promoting knowledge-sharing behaviors (Ridwan et al., 2022). Learning organizations help create a learning atmosphere of continuous communication and knowledge sharing among employees. Simultaneously, the establishment of the system can deepen and update knowledge among employees, increase their knowledge base, and increase their willingness to engage in knowledge sharing in the organization, thus promoting knowledge behaviors (Wang et al., 2022). In addition, its presence exudes psychological safety in the work environment, so employees are free to express themselves and try out new ideas. The organization has specific learning processes and practices that support individual and group learning and development, in which employees are more willing to share what they know with their colleagues (Korn et al., 2021). Based on these considerations, the following hypotheses are proposed:

*H1*: Learning organization system will positively influence knowledge sharing

### 2.2 Learning organization system and innovation performance

Innovation performance is the process by which employees purposefully propose, implement, and obtain ideas or results that benefit innovation within an organization (Peng et al., 2020). It is also the process by which employees purposefully create, introduce, and implement new ideas to enhance their individual, group, or organizational performance (Zhu et al., 2023). A high level of innovation performance not only helps in the development of the organization but also enhances its competitiveness (Wan and Chen, 2012). Moreover, employees’ innovation performance will contribute to the overall innovation of the organization to a certain extent, which will help the organization’s sustainable development and competitive advantage (Zhu et al., 2023). According to previous studies, psychological contracts (Wu et al., 2018), tacit knowledge sharing (Li and Tu, 2018), and distributive justice (Yoon and Lee, 2014) were found to have significant positive effects on innovation performance.

A learning organization system can create a knowledge management mechanism to help employees acquire, share, and utilize knowledge as a method of learning, which stimulates their ideas and innovative capabilities to improve innovation performance (Purwanti and Rizky, 2012). Its emergence has moderated individual and collective learning, thereby promoting new ideas. Therefore, continuous learning by individuals and teams stimulates creativity, which in turn improves innovation and enhances employees’ innovation performance (Fanbasten, 2014). It also facilitates learning and personal development opportunities for all employees while supporting continuous organizational transformation, enabling employees to enhance self-innovation through ongoing learning and thereby improving performance (Laksono, 2023). A learning organization system improves employee performance, reduces knowledge acquisition costs, motivates continuous knowledge updating, and stimulates competence and innovation, thereby enhancing innovation performance (Purwanti and Rizky, 2012). The existence of a learning organization system will help employees in the organization update their knowledge in the form of continuous learning, which will motivate them to be innovative and improve their innovation performance (Laksono, 2023). Based on these considerations, the following hypotheses are proposed:

*H2*: Learning organization system will positively influence innovation performance

### 2.3 Knowledge sharing and innovation performance

Employees interact and communicate with different people in the process of knowledge sharing and these exchanges of ideas have been identified as potential sources of innovation, leading to superior organizational performance (Zhu et al., 2023). In organizations, employees can collaborate with others through knowledge sharing to innovatively solve problems and promote innovative performance (Peng et al., 2020). Moreover, knowledge-sharing behaviors help employees acquire knowledge better and facilitate the exchange of ideas with colleagues in the organization, leading to higher levels of innovation and improved innovation performance (Wan and Chen, 2012). When employees engage in higher levels of knowledge sharing, they communicate more fully, and the exchange of ideas during mutual support is more likely to generate new insights, thereby enhancing innovation performance (Wu et al., 2018). In addition, in an organization, employees’ knowledge-sharing behaviors improve knowledge retention, reduce acquisition costs, and accelerate new knowledge accumulation, thereby enhancing innovation performance (Cheng and Wang, 2022). Based on these considerations, this study proposes the following hypotheses:

*H3*: Knowledge sharing will positively influence innovation performance.

### 2.4 The mediating effect of knowledge sharing

In the process of technological integration, organizations need to improve through learning. A learning organization system supports employee learning by providing material and spiritual protection, stimulating knowledge-sharing willingness and behaviors, enabling employees to leverage their strengths and enhance self-innovation performance (Wu et al., 2018). A learning organization system enables employees to acquire new knowledge through continuous learning and knowledge sharing, which the organization can leverage to promote innovation, enhance employee innovation capability, and improve innovation performance (Fanbasten, 2014). A learning organization system helps organizations motivate employees and promote technical knowledge sharing, shaping a positive learning culture that drives continuous improvement and enhances innovation performance (Wu et al., 2018). Moreover, a learning organization system provides a favorable work environment that satisfies employees’ needs, leading to higher job satisfaction and a greater willingness to share knowledge, thereby improving performance (Laksono, 2023). In addition, it facilitates effective knowledge exchange and collaboration, enabling the generation of new knowledge that enhances employee innovation, effectiveness, and efficiency, ultimately promoting innovation performance (Laeeque et al., 2017). Based on these considerations, this study proposes the following hypotheses:

*H4*: Knowledge sharing will mediate the relationship between learning organizational system and innovation performance

### 2.5 The moderating effect of organizational cultural identification

This study emphasizes the moderating role of organizational cultural identification and argues that its emergence enhances the role of the learning organization system in the relationship between employees’ knowledge sharing behaviors. Therefore, employees’ knowledge sharing behaviors in Chinese SMEs are determined by the interaction between the learning organization system and organizational culture identification. Organizational cultural identification refers to the perception that employees are aware of their affiliation with the organization, which, in turn, leads them to modify their beliefs and behaviors to achieve organizational goals (Ding and Luo, 2012). It is also the extent to which employees share and accept the organization’s core values, beliefs, and behavioral norms (Chen and Huang, 2020). When employees identify with the organizational culture, they have a rational sense of contract and responsibility and clearly recognize their duties and obligations to work hard to achieve organizational goals and interests (Xie et al., 2023). Moreover, based on organizational cultural identification and a sense of belonging, employees exhibit behaviors that benefit the organization, actively participate in their work, and emotionally engage in it (Xie et al., 2023). According to prior research, organizational cultural identification has a significant positive impact on employees’ voice behavior (Xie et al., 2023), deep emotional behavior (Ding and Luo, 2012), and emotional commitment (Han and Wang, 2015).

Through learning systems, organizations can create a conducive environment for employee growth and foster an organizational learning culture. When employees feel supported and have access to learning opportunities, their organizational identification increases (Uludağ et al., 2023). Additionally, a learning organization system helps employees acquire knowledge and skills, improve learning ability, and grow alongside the organization, thereby fostering an organizational cultural identity rooted in shared beliefs and values (Wang and Chen, 2014). Moreover, A learning organization system makes employees feel supported in their learning, helping them better integrate into the organization, over time, employees develop resonance with the organization’s culture and values (Uludağ et al., 2023). Therefore, when employees and the organization share aligned cultures and values within a learning organization system, employees demonstrate a stronger sense of identification (Lingtao, 2023). In addition, the learning organization system aims to promote employee learning and development. Knowledge serves as a valuable intangible asset for creating and sustaining competitive advantage (Zhu et al., 2023). The establishment of a learning organization system promotes a culture of continuous learning and knowledge dissemination, which helps improve knowledge-sharing practices among employees (Laksono, 2023) and assists employees in achieving organizational goals. Organizational culture is considered an important factor in enhancing organizational performance, promoting innovation, and improving employee behavior (Lingtao, 2023). In organizations, higher organizational cultural identification arises when employees align with the organization’s values and beliefs, making them more willing to engage in open communication, collaboration, and knowledge sharing (Laksono, 2023). Organizational cultural identification promotes employee learning and development, makes employees feel valued and supported, increases self-motivation and satisfaction, and leads to a greater focus on knowledge sharing and innovation within the organization (Lingtao, 2023). In other words, when employees identify with the organizational culture of the organization, they are more willing to share their experiences, knowledge, and best practices with the organization and among their colleagues through knowledge sharing to help achieve the common goals of the organization. Therefore, learning organization systems encourage continuous learning while helping employees develop stronger organizational cultural identification and align with organizational values, which increases knowledge interactions and fosters knowledge-sharing behaviors (Laeeque et al., 2017). In other words, by creating a learning culture, providing resources, and building trust and support, a learning organization system fosters an environment that encourages learning and innovation, thereby enhancing employees’ identification with organizational culture and promoting active knowledge sharing. Grounded in social exchange theory, this supportive environment establishes a reciprocal dynamic: employees interpret organizational investment in their development as a cue to reciprocate through knowledge sharing, which in turn strengthens their organizational identification and sustains reciprocal behaviors, forming a virtuous cycle. Based on these considerations, this study proposes the following hypotheses:

*H5*: Organizational cultural identification has a positive moderating effect on the relationship between the learning organization system and knowledge sharing

## 3 Methods

### 3.1 Sample characteristics

This study is based on the employees of Chinese SMEs surveyed through an online questionnaire. Participants responded voluntarily and confidentially to the survey. The informed consent was obtained from all human research participants. A total of 300 data samples were collected from this questionnaire and used for empirical analysis. Regarding the demographic characteristics of this study, 158 (52.7%) patients were male, and 142 (47.3%) were female.

Regarding age, 0(0%) participants were 19 years old, 107(35.7%) were 20 to 30 years old, 93(31.0%) were 31 to 40 years old, 62(20.7%) were 41 to 50 years old, 37(12.3%) were 51 to 60 years old, and one (0.3%) was 60 years or older.

Regarding education, 149(49.7%) were junior college graduates or lower, 106(35.3%) were college graduates, 32(10.7%) had master’s degrees, and 13(4.3%) were doctors.

Regarding Service Years, 29(9.7%) people had worked for less than 1 year, 59(19.7%) for less than one to 3 years, 38(12.7%) for less than three to 5 years, 24(8.0%) for less than five to 7 years, and 150(50.0%) people had worked for seven or more years.

Regarding enterprise type, 28 (9.3%) worked in the construction industry, 54 (18.0%) in the food service industry, 16 (5.3%) in finance, 21 (7.0%) of the individuals worked in the education sector, five (1.7%) in the medical industry, eight (2.7%) in the information technology industry, and 168 (56.0%) in various other fields.

### 3.2 Measurement

A learning organization system refers to the characteristics of an organization system that should be established and possessed by a learning organization to maintain sustainable survival and healthy and harmonious development in a complex and changing environment (Chen, 2008). To measure Chinese SME learning organization systems, this study used a tool mentioned in Chen (2008), which consists of 25 items. Sample items included “The organization employs certain methods to promote continuous learning and innovation among its employees” or “The enterprise recognizes and rewards the contribution of individual employees”. Knowledge sharing refers to expanding the process of knowledge exchange and discussion between knowledge owners and recipients through various channels within or between organizations (Wang et al., 2023). To measure Chinese SMEs’ knowledge sharing, this study used the tool mentioned by Bock and Kim (2002). The measurement tool consists of five items. Sample items included “’ I will share my knowledge with more organizational members.” or “I will always provide my knowledge at the request of other organizational members”.

Organizational cultural identification refers to the process of building an organizational culture through the participation of internal and external stakeholders, fully considering their interests and needs, and finally being formed and recognized by employees (Liang, 2013). To measure Chinese SMEs’ organizational cultural identification, this study used a tool mentioned in the studies of Chen and Zhang (2009). The measurement tool consisted of 20 items. Sample items included “I know exactly what our company’s culture is all about.” or “I appreciate our company’s cultural values”.

**Figure 1:**
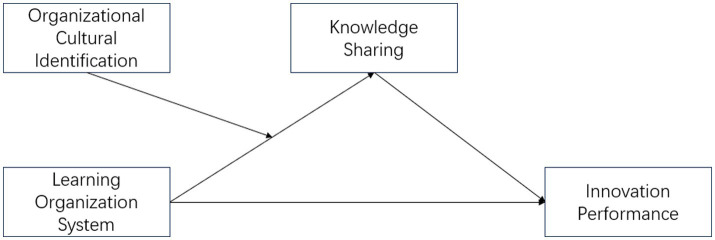
Research model.

Innovation performance is the overall output produced by an organization through processes or activities related to innovation. It refers to the extent to which members of the organization achieve job performance efficiently and effectively (Lee et al., 2016). To measure Chinese SMEs’ innovation performance, this study uses the tool mentioned by Janssen and Van Yperen (2004). The measurement tool consists of nine items. Sample items included “Creating new ideas for improvements.” or “Mobilizing support for innovative ideas”. All items were measured on a 7-point Likert scale (ranging from 1 = Strongly Disagree to 7 = strongly agree) (Figure 1).

## 4 Results

### 4.1 Confirmatory factor analysis and reliability analysis

In this study, the practicability of the different data models was verified using confirmatory factor analysis (Presson et al., 1997). The results of the confirmatory factor analysis were as follows: The absolute fit indices were *X*^2^(p) = 4292.218(0.000), *X*^2^/df = 3.036, and RMSEA = 0.083. The RMSEA is indeed a ‘badness of fit’ index, with values very close to 0 indicating an almost perfect fit and with greater RMSEA indicating a worse fit. For the RMSEA, values less than 0.05 reflect a small approximation error, values between 0.05 and 0.08 reflect an acceptable error of approximation, and values greater than 0.10 constitute a poor fit of the model (Browne and Cudeck, 1992). Moreover, RMSEA, as an absolute fit index, indicates good model fit when its value is less than 0.08, and acceptable fit when it falls between 0.08 and 0.10 (MacCallum et al., 1996). Accordingly, the RMSEA value of 0.083 obtained in this study falls within the “acceptable fit” range. Second, the incremental fit indices were IFI = 0.902 and CFI = 0.901. The parsimonious adjusted indices were PNFI = 0.711 and PGFI = 0.561. Therefore, upon comprehensive evaluation with other fit indices, multiple indicators converge to support the conclusion of acceptable model fit. Although the RMSEA value marginally exceeds the stringent cutoff of 0.08, the overall model fit in this study remains within an acceptable range when considered in conjunction with the full array of fit indices and theoretical foundations.

This study analyzed the average variance extracted (AVE) and composite reliability (C. R) values. Regarding average variance extracted (AVE), the learning organization system was 0.673, knowledge sharing was 0.808, organizational cultural identification was 0.773, and innovation performance was 0.633; these values were all greater than 0.5.

Regarding composite reliability (C. R), the learning organization system was 0.973, knowledge sharing was 0.934, organizational cultural identification was 0.977, and innovation performance was 0.907; all these values were greater than 0.7. The measurement has significant validity if the AVE of the variables is higher than 0.5 and the CR is higher than 0.7.

Reliability analysis refers to the method of measuring the internal consistency of scale items (Qureshi et al., 2023). Therefore, this study also analyzed the Cronbach’s alpha. For values of *α* Cronbach’s, learning organization system = 0.984, knowledge sharing = 0.953, organizational cultural identification = 0.986 and innovation performance = 0.981; reliability analysis has significant validity if Cronbach’s of variables is higher than 0.7. Table 1 presents the results of the study.

**Table 1:** The result of confirmatory factor analysis and reliability analysis.

Variables	Estimate		S. E.	C. R.	*p*	Standardized regression weights	AVE	C. R	Cronbach’s alpha
Learning organization system (A)	A25	1				0.852	0.673	0.973	0.984
	A24	1.027	0.034	29.944	***	0.855			
	A23	1.017	0.033	31.221	***	0.836			
	A22	1.048	0.04	26.232	***	0.843			
	A21	1.039	0.044	23.682	***	0.827			
	A20	1.043	0.042	24.587	***	0.864			
	A19	1.038	0.041	25.123	***	0.88			
	A18	1.082	0.039	27.765	***	0.91			
	A17	1.079	0.044	24.275	***	0.869			
	A16	1.036	0.043	24.219	***	0.868			
	A15	1.033	0.043	23.982	***	0.867			
	A14	1.018	0.041	24.671	***	0.876			
	A13	0.954	0.043	22.145	***	0.841			
	A12	1.011	0.042	23.884	***	0.866			
	A11	0.959	0.042	22.637	***	0.848			
	A10	0.982	0.046	21.137	***	0.825			
	A9	0.868	0.041	21.131	***	0.824			
	A8	0.934	0.043	21.535	***	0.831			
	A7	0.81	0.047	17.313	***	0.748			
	A6	0.8	0.048	16.761	***	0.735			
	A5	0.926	0.052	17.754	***	0.758			
	A4	0.905	0.053	17.14	***	0.744			
	A3	0.784	0.05	15.653	***	0.708			
	A2	0.84	0.055	15.405	***	0.701			
	A1	0.858	0.057	14.945	***	0.688			
Knowledge sharing (B)	B1	1				0.889	0.808	0.934	0.953
	B2	1.077	0.037	29.357	***	0.88			
	B3	1.15	0.04	28.486	***	0.912			
	B4	1.112	0.039	28.431	***	0.912			
	B5	1.139	0.042	27.302	***	0.901			
Organizational cultural identification (C)	C20	1					0.773	0.977	0.986
	C19	0.919	0.034	26.939	***	0.859			
	C18	1.016	0.049	20.585	***	0.832			
	C17	1.14	0.042	27.389	***	0.782			
	C16	1.193	0.046	26.035	***	0.9			
	C15	1.119	0.039	28.578	***	0.889			
	C14	1.14	0.047	24.466	***	0.913			
	C13	1.173	0.047	24.719	***	0.867			
	C12	1.242	0.043	28.808	***	0.869			
	C11	1.156	0.039	29.608	***	0.915			
	C10	1.159	0.04	29.048	***	0.922			
	C9	1.174	0.04	29.008	***	0.917			
	C8	1.103	0.048	23.17	***	0.916			
	C7	1.186	0.04	29.999	***	0.849			
	C6	1.2	0.04	30.305	***	0.925			
	C5	1.029	0.04	25.985	***	0.928			
	C4	1.039	0.043	24.206	***	0.885			
	C3	1.033	0.052	19.839	***	0.863			
	C2	1.056	0.042	24.899	***	0.794			
	C1	1.06	0.043	24.698	***	0.872			
Innovation performance (D)	D1	1				0.82	0.633	0.907	0.981
	D2	0.966	0.033	29.41	***	0.781			
	D3	1.003	0.033	30.068	***	0.795			
	D4	1.069	0.036	29.316	***	0.794			
	D5	0.985	0.032	30.666	***	0.801			
	D6	0.987	0.041	23.889	***	0.786			
	D7	0.957	0.037	25.967	***	0.8			
	D8	1.077	0.047	23.076	***	0.811			
	D9	0.944	0.039	24.334	***	0.773			
Model Fit Index		X^2^(*p*) = 4292.218(0.000), *X*^2^/df = 3.306, RMSEA = 0.083, IFI = 0.902, CFI = 0.901, PGFI = 0.561, PNFI = 0.711							

### 4.2 Descriptive statistics and correlation analysis

Table 2 shows the descriptive statistics and correlation analysis. Descriptive statistical analyses included mean and standard deviation (SD). The means for learning about the organizational system, knowledge sharing, organizational cultural identification, and innovation performance were 5.862, 5.763, 5.712, and 5.687, respectively. In addition, the SDs for the learning organization system, knowledge sharing, organizational cultural identification, and innovation performance are 1.031, 1.096, 1.111, and 1.169, respectively.

**Table 2:** The results of descriptive statistics and correlation analysis.

Variables	Mean	Standard deviation	Learning organization system	Knowledge sharing	Organizational cultural identification	Innovation performance
Learning organization system	5.862	1.031	–			
Knowledge sharing	5.763	1.096	0.782***	–		
Organizational cultural identification	5.712	1.111	0.836***	0.801***	–	
Innovation performance	5.687	1.169	0.785***	0.733***	0.870***	–

***p < 0.001; **p < 0.01; *p < 0.05.

To verify the correlation among variables, this study conducted a correlation analysis, the results of which are summarized as follows: the learning organization system was positively associated with knowledge sharing (*r* = 0.782, *p* < 0.001), organizational cultural identification (*r* = 0.836, *p* < 0.001), and innovation performance (*r* = 0.785, *p* < 0.001). Knowledge sharing was positively associated with organizational cultural identification (*r* = 0.801, *p* < 0.001) and innovation performance (*r* = 0.733, *p* < 0.001). Moreover, organizational cultural identification was positively associated with innovation performance (*r* = 0.870, *p* < 0.001).

### 4.3 Path analysis

SPSS Process Model 4 was used to analyze the mediation effect of knowledge sharing. The results show that the learning organization system has a positive impact on knowledge sharing (Estimate = 0.831, *p* < 0.001) and has a positive impact on innovation performance (Estimate = 0.618, *p* < 0.001). Additionally, the results show that knowledge sharing has a positive impact on innovation performance (Estimate = 0.326, *p* < 0.001). Therefore, Hypotheses 1, 2, and 3 were supported.

Hypothesis 4 establishes that knowledge sharing mediates the relationship between the learning organization system and innovation performance. The indirect effect was 0.271. The bootstrapped confidence intervals were Boot LLCI = 0.139 and Boot ULCI = 0.428, as 0 was not included between Boot LLCI and Boot ULCI. These results indicate that the mediating effect of knowledge sharing was significant. This finding suggests that a learning organization system increases innovation performance through knowledge sharing. Thus, Hypothesis 4 is supported. Table 3 presents the results of the path analysis.

**Table 3:** The results of process model 4.

Path			Estimate	S. E.	*t*	*p*	LLCI	ULCI
Learning organization system	→	Knowledge sharing	0.831	0.038	21.682	0.000	0.755	0.906
Learning organization system	→	Innovation performance	0.618	0.062	9.940	0.000	0.496	0.741
Knowledge sharing	→	Innovation performance	0.326	0.058	5.570	0.000	0.211	0.441

Indirect effect(s) of X on Y				
Indirect effect	Effect	Boot SE	Boot LLCI	Boot ULCI
Learning organization system → Knowledge sharing → Innovation performance	0.271	0.073	0.139	0.428

### 4.4 Moderating effect of organizational cultural identification

**Figure 2:**
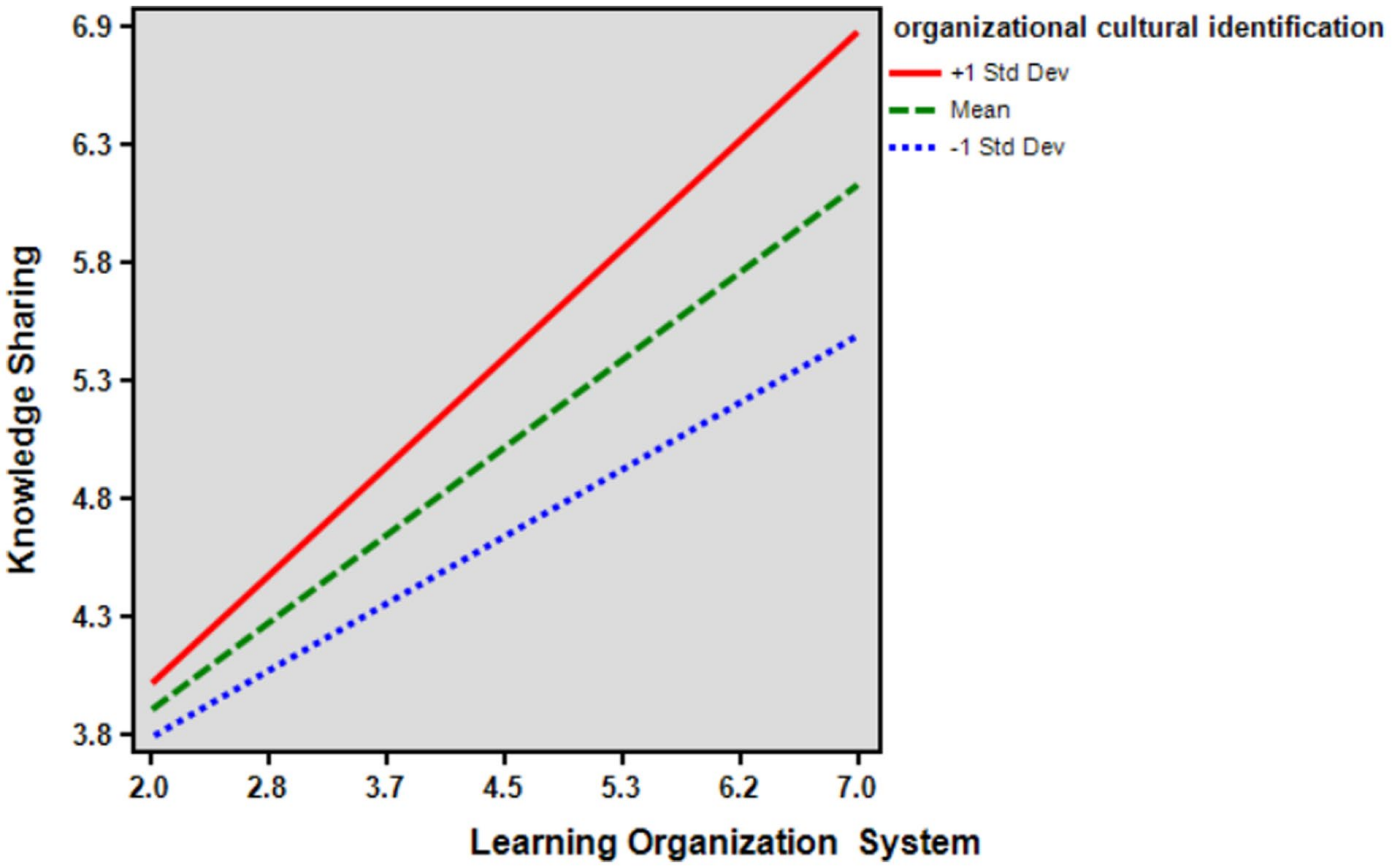
The moderating effect of organizational cultural identification.

Hypothesis 5 established that organizational cultural identification moderates the effect of the learning organization system on knowledge sharing. The results showed that organizational cultural identification significantly moderated the effect of the learning organization system on knowledge sharing (*β* = 0.126, *p* < 0.001). This means that the higher the organizational cultural identification, the greater the impact of the organizational system on knowledge sharing. Therefore, Hypothesis 5 is supported. Therefore, the results show that the interaction between organizational cultural identification and the learning organization system leads to a higher degree of knowledge sharing (Figure 2; Table 4).

**Table 4:** The result of moderation.

Dependent variable: knowledge sharing							
	Model 1		Model 2		Model 3		VIF
	*β*	*t*	*β*	*t*	*β*	*t*	
Learning organization system (A)	0.782^***^	21.683	0.375^***^	6.301	0.430^***^	7.112	3.569
Organizational cultural identification (B)			0.488^***^	8.201	0.490^***^	8.397	3.320
Interaction					0.126^**^	3.489	1.128
*R^2^*(Adjusted *R^2^*)	0.612 (0.611)		0.684 (0.682)		0.696 (0.693)		
*⊿R^2^*(⊿Adjusted *R^2^*)	–		0.072 (0.071)		0.012 (0.011)		
*F*	470.141^***^		320.962^***^		226.079^***^		

***p < 0.001; **p < 0.01; *p < 0.05.

## 5 Discussion

Previous studies (e.g., Lungit et al., 2020; Basu and Ray, 2013; Hu and Wang, 2008) have confirmed the impacts and effects of the establishment of a learning organization system on an organization, emphasizing its importance. It is considered that the establishment or improvement of the learning organization system is an important task to achieve sustainable development of the organization and improve the future competitive advantage of the enterprise. Although this has been confirmed, relatively little research has been conducted on learning organization systems. Therefore, we explore the impact of learning organization systems on organizations and employees in the context of Chinese SMEs. Innovation performance is defined as the overall performance of an organization or an individual’s innovation activities and efficiency. We present innovation performance as a product of establishing a learning organization system. The relationship between the learning organization system and innovation performance was clarified. The results of this study show that the establishment of a learning organization system aims to help employees learn continuously through a learning mechanism and organizational culture, adapting to and innovating the organizational form, promoting the enhancement and updating of employees’ knowledge, and prompting them to share their knowledge and communicate effectively with each other while being supported by resources to enhance the level of innovation performance. In other words, improvement in employees’ knowledge-sharing behaviors in the organization will also increase the level of innovation performance. The positive correlation between employees’ knowledge sharing and innovation performance is stronger. In addition, through a moderated model, we verified whether the improvement in knowledge-sharing behavior depends on the interaction between the learning organization system and organizational cultural identification. This study provides insights and directions for future research on the organizational sustainability of Chinese SMEs. The findings are summarized as follows.

### 5.1 Theoretical implications

The main contribution of this study is that it explores and determines through empirical analysis how a learning organization system improves employees’ innovation performance. This study focuses on the direct impact of a learning organization’s system on innovation performance. It also specifically examines the key variables that play a role in the learning organization system by inducing innovation performance.

First, the learning organization system has a positive effect on knowledge sharing. This indicates that the higher the level of the learning organization system, the higher the level of knowledge sharing. The learning organization system establishes a symbiotic relationship with the environment and builds an organizational community with related subjects to achieve information exchange and knowledge acquisition. This leads to the rapid flow and sharing of knowledge within the organization, thus achieving effective organizational learning (Zhang and Cui, 2008). Moreover, a learning organization system resets organizational structure, reduces distance and conflict between departments, and eliminates boundaries, thereby facilitating knowledge flow and increasing knowledge-sharing opportunities (Fan, 2003). Specifically, it fosters a learning culture and capability by promoting knowledge creation, acquisition, integration, and application. This reduces employees’ knowledge acquisition costs, accelerates knowledge flow and transformation through effective communication and sharing, and encourages interaction and cooperation among members to achieve organizational development.

Second, the results show that the learning organizational system has a positive effect on innovation performance. This indicates that the higher the level of the learning organization system, the higher the level of employees’ innovation performance. Innovation performance is not only a result of proactive behaviors that are actively adopted by employees but also a manifestation of processes and ideas that create novel and valuable products for the organization (Zhao et al., 2024). In an organization, the establishment of a learning organization system continuously encourages employees to engage in learning so that they can adapt to external changes and remain competitive (Kim et al., 2024), thus enhancing the continuous improvement of knowledge and ideas. In other words, the establishment of a learning organization system enhances the flexibility of the organizational structure through continuous learning, allowing the organization to respond rapidly to the market, stimulate innovation, and prepare for improved performance (Zhang and Cui, 2008). Therefore, the learning organization system provides good support and assistance to the innovation of the organization’s employees and helps the organization maintain its competitive advantage to continuously create value.

Third, knowledge sharing positively affects innovation performance. This suggests that the higher the level of knowledge sharing among employees, the higher the innovation performance. In organizations, employees’ knowledge-sharing behavior can lead to intergroup communication, sharing expertise, views, and ideas, and promoting mutual learning among employees (Zamiri and Esmaeili, 2024). Moreover, knowledge sharing can effectively integrate and update employees’ knowledge and promote organizational performance (Li and Qi, 2023). Specifically, knowledge sharing promotes the dissemination of technical knowledge, rapid technology diffusion, and integration of complementary technologies, leading to improved innovation performance (Wu et al., 2018). Therefore, when knowledge sharing within an organization is effective, employees can easily access and utilize various resources and experiences, facilitating the generation of stimulating innovative ideas and solutions, which in turn drive innovation and improve individual performance.

Fourth, knowledge sharing mediates the relationship between the learning organization system and innovation performance. This suggests that a learning organization system can impact innovation performance through employees’ knowledge-sharing behaviors. Knowledge sharing, transfer, innovation, and application are inseparable from organizational learning (Zhang and Cui, 2008). A learning organization system deepens employees’ awareness of organizational learning, emphasizing continuous learning to enhance motivation and performance through knowledge and experience sharing (Ambarwati et al., 2020). A learning organization system provides employees with a comprehensive learning platform, enabling them to access rich knowledge resources from both internal and external sources. By advocating knowledge dissemination, it enhances employees’ awareness and willingness to share knowledge, stimulating innovative thinking in the process. This allows innovations to be realized and transformed into innovative performance more quickly, providing a foundation for organizational development.

Finally, this study verifies the moderating role of organizational cultural identification between the learning organization system and knowledge sharing. The findings of this study are consistent with the core tenets of social exchange theory, confirming that a supportive learning environment can stimulate employees’ sense of reciprocal obligation and thereby promote knowledge sharing behaviors. The results show that organizational cultural identification has a positive moderating effect on the learning organization system and knowledge sharing. This suggests that the higher the interaction between employees’ organizational cultural identification and the learning organization system, the higher the knowledge sharing behavior. Organizational culture, the values and beliefs shared by organizational members, influences employee behavior in the organizational learning process (Wang and Fang, 2013). An increase in the level of identification is seen when the level of the cultural base of the organization matches that of the individual (Uludağ et al., 2023). Therefore, employees with higher organizational cultural identification have an appreciative attitude toward the organization, derive satisfaction from working with attitudinal and behavioral congruence, and are more emotionally invested in the organization (Han and Wang, 2015). The systematization of learning organizations will form a learning organizational culture within the organization and promote learning and knowledge accumulation, motivate employees, and encourage innovation; in such an environment, employees will feel that their value is enhanced, which will increase their organizational cultural identification (Lingtao, 2023). Therefore, a learning organization system helps establish a learning culture that motivates continuous learning. When employees see their beliefs align with organizational values and their contributions are recognized, trust in the organization and colleagues grows, promoting knowledge sharing.

### 5.2 Practical implication

First, to play the human subjective initiative as the core performance of organizational management, social thinking gradually changed from linear thinking to systematic thinking and creative thinking, and the economic characteristics of the times put forward higher requirements on the knowledge levels of individuals and enterprises (Yu, 2007). The learning organization system is an important organizational management system for enterprise transformation. Its emergence creates a set of mechanisms and practices in the organization that form a culture that encourages and supports continuous learning habits, creates an enabling environment for collective learning through continuous learning and improvement, and ultimately leads to the development of a shared vision (Palos and Veres Stancovici, 2016). Therefore, in organizational management practices, organizations should recognize the necessity and importance of the existence of a learning organization system and continuously assess and improve it to ensure that it is aligned with organizational goals. Employees need to continually improve their effectiveness and sustainability for the future development and competitive advantage of the organization. Therefore, in organizational management practice, managers should fully recognize the strategic value of building a learning organization system and regard it as a core pillar for enhancing organizational resilience and sustained competitiveness. Organizations need to regularly evaluate the operational effectiveness of the learning system and make dynamic adjustments to ensure that it remains highly aligned with organizational strategic goals while flexibly responding to challenges posed by changes in the external environment. For employees, a learning organization not only provides a platform for knowledge renewal and capability enhancement but also requires them to proactively improve work efficiency and cultivate awareness of sustainable development to meet the evolving demands of the organization. Only by closely integrating individual continuous learning with organizational strategic direction can the full potential of the learning system be unleashed, enabling the organization to achieve long-term development and competitive advantage in a complex and dynamic market environment. Therefore, the findings of this study possess a certain degree of external validity. Organizations may, in future practice, draw upon the insights of this study in light of their specific characteristics, integrating the concept of a learning organization into core areas such as talent development, performance management, and organizational culture construction, thereby providing a basis for advancing the deep integration of theory and practice.

Second, knowledge sharing helps organizations and employees improve their understanding, skills, and problem-solving abilities, which, in turn, contribute to the continuous growth and progress of the firm (Zamiri and Esmaeili, 2024). Moreover, it can improve an organization’s resilience and innovation (You et al., 2005). In addition, the dissemination and application of knowledge by employees within an organization can be innovative, improve decision-making, and foster the development of collective intelligence to enhance the sustainable development of the organization (Zamiri and Esmaeili, 2024). Therefore, in management practice, organizations should actively advocate and support the culture of knowledge sharing, establish open communication channels and sharing platforms through the learning organization system, and encourage employees to share their professional knowledge, experience, and other behaviors; at the same time, establish a feedback mechanism in order to understand the effect of knowledge sharing in a timely manner, so as to make adjustments and improvements, and thus to promote learning and innovation in the organization. Through the aforementioned measures, organizations can effectively break down barriers and boundaries between departments, facilitate the flow and transformation of tacit knowledge, and form a virtuous cycle of continuous learning and innovation. When knowledge sharing becomes an institutionalized mechanism within the organization, employees will participate more actively in collaborative and innovative activities, thereby enhancing the organization’s overall learning capacity and adaptability. This provides a valuable reference for building an efficient learning organization system.

Third, cultural factors are necessary to facilitate the organizational learning process (Lau et al., 2017). Organizational cultural identification is an intrinsic motivation that influences employees’ emotional work and drives deep-seated behaviors (Ding and Luo, 2012). Employees’ cultural adoption of the organization’s values, beliefs, etc., is reflected in the extent to which their behaviors are aligned with those of the organization (Uludağ et al., 2023). Therefore, in management practices, organizations should pay attention to and shape the organizational culture to promote the cohesion and efficiency of employees’ organizational cultural identification and to emphasize the consistency of values between the organization and its employees through the establishment of a positive work environment. Leaders, as creators and distributors of organizational culture, should focus on their behavior in the organization to ensure that they are consistent with the organization’s values, thus enhancing employees’ identification with the organization’s mission and vision. Motivate employees to be more active in supporting the development of an organization. When organizational culture aligns deeply with employees’ personal values, employees will demonstrate higher levels of organizational citizenship behavior and extra-role performance, thereby providing solid talent support for the organization’s sustained innovation and strategic transformation.

Fourth, innovation performance is key to gaining a competitive advantage in highly volatile environments (Wijaya et al., 2023). The higher the productivity and performance of employees, the more an organization can improve its development and economy; employee performance is the output produced by employees to achieve organizational goals (Ambarwati et al., 2020). Therefore, in organizational management practices, organizations should pay attention to the innovation performance of employees and create a culture and system that supports innovation through the learning organizational system, which encourages employees to come up with new perspectives and try out new ideas. Simultaneously, leaders should empower employees with autonomy and decision-making power to stimulate their creativity and innovation potential to promote employee performance for an organization’s continuous innovation and performance improvement to improve help. Through the aforementioned mechanisms, a learning organization system can establish a virtuous cycle driven by innovation within the organization, enabling employees to internalize innovative behaviors as a normalized way of working under the dual influence of institutional support and cultural guidance. This organizational design, grounded in the synergy between empowerment and culture, not only enhances individual-level innovation performance but also promotes a systemic leap in the organization’s overall innovation capability through the spillover effects of knowledge sharing and team collaboration.

### 5.3 Limitations and future research

Although this study validates the impact of knowledge sharing on the relationship between the learning organization system and innovation performance and the moderating aspects of organizational cultural identification provide noteworthy contributions, it has some limitations. Specifically, it is expressed as.

First, it examines the role of organizational systems in organizations in the Chinese context. Considering differences in geography and culture, similar studies are necessary to understand the situation in other countries. This is because it is important to conduct an empirical analysis of learning organization systems from different countries and cultural contexts to determine whether similar results can be derived (Jin et al., 2023). Future research should compare the findings from different regions to better understand the universality and variability of this phenomenon.

Second, this study focuses on learning organization systems. Organizational learning is the capability advocated by the creation of a learning organization system. It refers to the process by which members of an organization continuously acquire knowledge, improve their behaviors, and optimize the organization’s system so that the organization can maintain sustainable survival and healthy and harmonious development in the changing internal and external environments (Chen, 2008). Organizational learning can improve the common concepts and behaviors of an organization, which in turn improves its performance and thus enhances its future development (Yu et al., 2006). Therefore, it is necessary to begin with the organizational learning aspect in future research to explore its importance and impact on organizations and their employees.

Third, Innovation performance is divided into individual and organizational levels. This study focused on employee innovation performance at the individual level. Innovation performance is not only of great practical significance to the survival and development of enterprises but has also gradually become a common focus of organizational learning and innovation theory research (Sun and Liu, 2022). Therefore, future research should conduct more in-depth studies from the perspective of organizational innovation performance to provide useful support for the future development of organizations.

Fourth, the questionnaire used in this study was not deliberately categorized, and all variables were assessed by employees themselves. This single-source data collection may inflate the correlations among variables, posing a risk of common method bias (CMB). Future research could improve questionnaire administration by, for example, having employees evaluate organization-related issues while organizational leaders assess employees’ attitudes, behaviors, and performance (Jin et al., 2023). Such multi-source data collection would enhance the validity and persuasiveness of the findings.

Fifth, in this study, the RMSEA for the structural equation model was 0.083, slightly above the commonly recommended threshold of 0.08. fall within the acceptable range. This suggests room for further improvement. Future research could enhance the robustness and generalization of the model fit by employing larger sample sizes, streamlining measurement scales, utilizing longitudinal data, or conducting cross-validation across different contexts.

Finally, this study employed a cross-sectional design, in which all variables were measured at a single point in time. As a result, the findings have certain limitations in terms of inferring causal relationships and long-term effects. Cross-sectional data can only reflect the relationships among variables at a specific moment and cannot capture the dynamic evolution processes at the individual or group level within a learning organization system. In contrast, longitudinal research designs can track changes in the same subjects over multiple time points, offering greater insight into the sustained impact of learning organizations on employee growth, team learning, and organizational adaptability. Moreover, longitudinal data provide stronger evidence for examining interrelationships and even causal linkages among variables. Additionally, longitudinal approaches help control for individual heterogeneity and mitigate issues such as common method bias that are prevalent in cross-sectional designs. Therefore, future research should consider adopting a longitudinal design to collect data across multiple time points, tracing the trajectory of learning organization practices over time. By comparing the results with those of the present study, a more comprehensive and in-depth understanding of the long-term effects and underlying mechanisms of learning organization systems can be achieved.

## Data Availability

The datasets presented in this article are not readily available because the datasets generated and/or analyzed during the current study are available from the corresponding author upon reasonable request. Requests to access the datasets should be directed to Xiu Jin, soohua1005@gachon.ac.kr.
